# Pre-treatment inflamed tumor immune microenvironment is associated with FOLFIRINOX response in pancreatic cancer

**DOI:** 10.3389/fonc.2023.1274783

**Published:** 2023-11-23

**Authors:** Zachary Gao, Sung Wook Kang, Derek Erstad, Joseph Azar, George Van Buren, William Fisher, Zequn Sun, Mark P. Rubinstein, Hyun-Sung Lee, E. Ramsay Camp

**Affiliations:** ^1^ Michael E. DeBakey Department of Surgery, Baylor College of Medicine, Houston, TX, United States; ^2^ Department of Surgery, Dan L. Duncan Comprehensive Cancer Center, Houston, TX, United States; ^3^ Systems Onco-Immunology Laboratory, David J. Sugarbaker Division of Thoracic Surgery, Michael E. DeBakey Department of Surgery, Baylor College of Medicine, Houston, TX, United States; ^4^ Department of Surgery, Michael E. DeBakey VA Medical Center, Houston, TX, United States; ^5^ The Pelotonia Institute for Immuno-Oncology, Ohio State University Comprehensive Cancer Center, Columbus, OH, United States; ^6^ Department of Preventative Medicine, Northwestern University Clinical and Translational Sciences Institute, Chicago, IL, United States

**Keywords:** PDACpancreatic ductal adenocarcinoma1, CIBERSORT2, FOLFIRINOX3, immunomodulator(s)4, RNA sequencing5

## Abstract

**Introduction:**

Pancreatic adenocarcinoma (PDAC) is an aggressive tumor with limited response to both chemotherapy and immunotherapy. Pre-treatment tumor features within the tumor immune microenvironment (TiME) may influence treatment response. We hypothesized that the pre-treatment TiME composition differs between metastatic and primary lesions and would be associated with response to modified FOLFIRINOX (mFFX) or gemcitabine-based (Gem-based) therapy.

**Methods:**

Using RNAseq data from a cohort of treatment-naïve, advanced PDAC patients in the COMPASS trial, differential gene expression analysis of key immunomodulatory genes in were analyzed based on multiple parameters including tumor site, response to mFFX, and response to Gem-based treatment. The relative proportions of immune cell infiltration were defined using CIBERSORTx and Dirichlet regression.

**Results:**

145 samples were included in the analysis; 83 received mFFX, 62 received Gem-based therapy. Metastatic liver samples had both increased macrophage (1.2 times more, p < 0.05) and increased eosinophil infiltration (1.4 times more, p < 0.05) compared to primary lesion samples. Further analysis of the specific macrophage phenotypes revealed an increased M2 macrophage fraction in the liver samples. The pre-treatment CD8 T-cell, dendritic cell, and neutrophil infiltration of metastatic samples were associated with therapy response to mFFX (p < 0.05), while mast cell infiltration was associated with response to Gem-based therapy (p < 0.05). Multiple immunoinhibitory genes such as ADORA2A, CSF1R, KDR/VEGFR2, LAG3, PDCD1LG2, and TGFB1 and immunostimulatory genes including C10orf54, CXCL12, and TNFSF14/LIGHT were significantly associated with worse survival in patients who received mFFX (p = 0.01). There were no immunomodulatory genes associated with survival in the Gem-based cohort.

**Discussion:**

Our evidence implies that essential differences in the PDAC TiME exist between primary and metastatic tumors and an inflamed pretreatment TiME is associated with mFFX response. Defining components of the PDAC TiME that influence therapy response will provide opportunities for targeted therapeutic strategies that may need to be accounted for in designing personalized therapy to improve outcomes.

## 1 Introduction

Overall survival for pancreatic adenocarcinoma (PDAC) remains dismal with 5-year survival less than 10% (1, 2). Effective conventional cytotoxic chemotherapeutic regimens are limited; however, combinations such as FOLFIRINOX or gemcitabine/nab-paclitaxel have demonstrated efficacy and can prolong survival for PDAC patients by months (3, 4). Immune checkpoint inhibitors (ICI) have had dramatic success in malignancies such as melanoma (5) non-small cell lung cancer (6), and biliary tract cancer (7). Unfortunately, ICIs as monotherapy have essentially failed in PDAC (8), prompting investigations into strategies to potentiate PDAC immunotherapy.

A wide body of preclinical data (9–16) supports the concept that chemotherapy favorably modifies the tumor immune microenvironment (TiME) through a variety of mechanisms. For example, one of the components of FOLFIRINOX, oxaliplatin, causes DNA damage (17) and can induce immunogenic cell death (ICD) via release of damage-associated molecular patterns in tumors, uptake of tumor debris and neoantigens by antigen-presenting cells, and ultimately, induction of an adaptive immune response and cytotoxic T cell activity (12, 13, 18, 19). Similarly, 5FU is thought to selectively kill myeloid-derived suppressor cells (MDSCs) to enhance T cell mediated anti-tumoral immunity (20). Results from phase III trials across a wide range of cancers have demonstrated that chemotherapy such as oxaliplatin combined with ICI (chemo-ICI) leads to improved overall survival and outcome compared with chemotherapy alone (21–35). Currently, the two main chemotherapy regimens for PDAC are FOLFIRINOX (5-fluorouracil, leucovorin, irinotecan, oxaliplatin) (3) and gemcitabine with nab-paclitaxel (4). These treatments have widespread applicability in the treatment of PDAC and are administered both as systemic chemotherapy in unresectable and metastatic PDAC (3, 36) as well as in a neoadjuvant fashion to improve cancer resectability and survival (37, 38). While FOLFIRINOX is typically favored as the initial chemotherapeutic strategy (3), it is associated with increased toxicity compared with Gem-based regimens (3, 4). In practice, there is currently no indications to guide clinicians in choosing between the two chemotherapy regimens beyond the patient’s performance status (39, 40). However, in the clinic, FOLFIRINOX delivery is associated with increased tumor-infiltrating CD8^+^ T lymphocytes (TILs), decreased circulating regulatory T cells (Tregs), and can increase tumoral PD-L1 expression (41–43). Thus, FOLFIRNOX holds potential to augment ICI therapy in PDAC patients. Studies of combination chemo-ICI in advanced PDAC patients demonstrate improved survival compared to chemotherapy alone (44). mRNA vaccines have also been used in combination with FOLFIRINOX and anti-PD1 therapy, demonstrating the presence of persistent vaccine-expanded tumor-specific T-cells (45). These recent developments underscore the importance of understanding the dynamic interplay of the PDAC TiME and chemotherapy.

Classically, PDAC has been described to have a “cold” TiME, including multiple immunosuppressive cell lines such as Tregs, MDSCs, and M2-phenotypic tumor-associated macrophages (TAMs) (46–52). However, a growing body of evidence supports that the PDAC TiME is heterogeneous, and represented by a diverse milieu of immune cell phenotypes (53). While such heterogeneity has been well-described across a variety of cancers and associated with survival, available data in PDAC is limited. For example, the Immunoscore, which is based on quantification of CD3+/CD8+ lymphocyte heterogeneity at the core and boundary of tumors (54), can outperform traditional TNM staging in predicting disease-free survival and overall survival in colorectal cancer (54, 55) and other cancers (56, 57). Recent investigations have also described significant differences in the TiME between metastatic and primary lesions (58–60). It has been shown that PD-L1 expression is decreased in immune cells of metastatic lesions of triple negative breast cancer (61) and differences exist in PD-1+ TIL infiltration between metastatic and primary lung cancer lesions (62).

The association between chemotherapy response and the PDAC TiME has not been well characterized and the influence of disease site has not been investigated thoroughly. We evaluated publicly available data from the COMPASS trial, a prospective study of treatment-naïve patients with a diagnosis of locally advanced or metastatic PDAC who had core needle biopsies obtained prior to treatment used for whole genome sequencing and RNA sequencing (63, 64). By analyzing the unique genomic dataset from patients with advanced disease, we investigated what molecular and cellular determinants are associated with chemotherapy response. A secondary goal was to characterize the TiME based on the primary versus metastatic site for PDAC. Considering the biologic differences between metastatic and primary lesions, as well as the established influence of chemotherapy on the TiME, we hypothesized that the components of the pre-treatment TiME would differ between metastatic and primary lesions and would be associated with therapy response and survival in a cohort of advanced PDAC patients. Going forward, these findings may have important implications for personalized therapies and for designing next-generation immunotherapy combination strategies.

## 2 Methods

### 2.1 COMPASS trial

**Figure 1:**
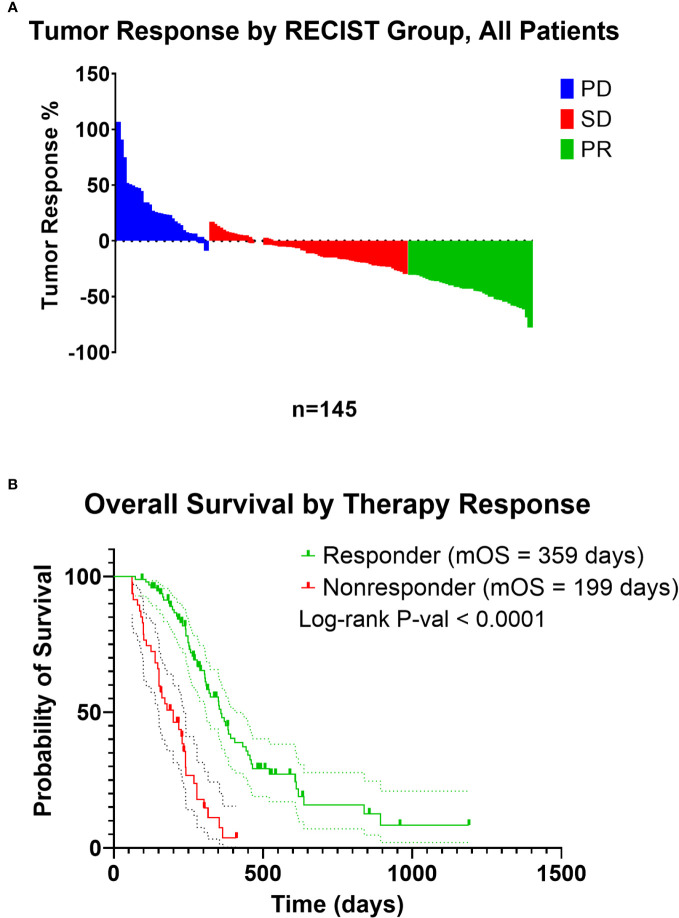
**(A)** Waterfall plot of tumor response of patients included in the analysis from the COMPASS trial. Patients were recoded from PD (Progressive Disease), SD (Stable Disease), and PR (Partial Response) to those with a decrease in tumor size on treatment as “responders”, and patients with an increase in tumor size on treatment as “nonresponders”. **(B)** Kaplan-Meier estimate of responders and nonresponders.

Institutional Review Board approval and written consent for the COMPASS trial (63, 64) was obtained from participating institutions (University Health Network, Toronto, Ontario, Canada; MUHC Centre for Applied Ethics, Montreal, Quebec, Canada; and Queen’s University Health Sciences and Affiliated Teaching Hospitals Research Ethics Board, Kingston, Ontario, Canada) (63, 64), and a data use agreement was completed by Baylor College of Medicine with the Ontario Institute for Cancer Research for use of the data within this study. Briefly, image-guided percutaneous core needle biopsies were obtained, and patients then received modified FOLFIRINOX (mFFX), gemcitabine/nab-paclitaxel, or a combination of these along with investigational drugs as standard first line therapy and had therapy response data by RECIST 1.1 (65). For our analysis, chemotherapy response data was defined based on tumor size change to therapy. “Responders” were patients whose measured tumor decreased in size, while “nonresponders” no change or an increase in tumor size while on therapy (**Figure 1A**). Patients included in our subsequent analysis were comprised of those who had a confirmed diagnosis of PDAC, had a biopsy obtained from either the liver or pancreas, had longer than 30-day survival from time of trial enrollment, had received at least one cycle of either mFFX or gemcitabine-based (Gem-based) therapy as treatment, and had RECIST data available for evaluation of therapy response.

### 2.2 Immunomodulatory differential gene expression analysis

Raw count data of RNA sequencing data of patients included in the COMPASS trial were downloaded from EGAD00001004548 (https://ega-archive.org/datasets/EGAD00001004548) and EGAD00001006081 (https://ega-archive.org/datasets/EGAD00001006081). Gene quantification was performed by TPMCalculator (66) and using GENCODE Human Release 43 version of gene annotation GTF file (67). As the immunomodulatory genes which influence immune cell infiltration into the TiME (68) can mediate chemoresistance to gemcitabine (69, 70) or platinum-based therapies (71), we evaluated the immunomodulatory genes within these samples as well. Tumor-Immune System Interactions Database (TISIDB) is an online repository of integrated data of tumor-immune interactions (72), including a curated list of genes encoding immunomodulators based on data from 30 non-hematologic cancer types from The Cancer Genome Atlas (TCGA). The raw count data were processed using edgeR v3.42.4 (73) by filtering to remove lowly expressed genes using the “filterByExpr” function, normalization by trimmed mean of M values (74), and dispersion estimation using the negative binomial distribution method. Differential gene expression analysis was calculated using the quasi-likelihood pipeline with a nominal log fold change threshold of 0.5 and a false discovery rate correction (73) set at a nominal value of 0.05 using genes of interest were obtained from TISIDB. Immunomodulatory genes were divided into immunoinhibitory, immunostimulatory, and MHC genes.

### 2.3 In silico cytometry based on transcriptomics

The leukocyte composition of each sample was then characterized as an immune cellular fraction using CIBERSORTx, which estimates proportions of immune cell populations from deconvoluted bulk transcriptomic data (67). CIBERSORTx analysis was performed using the following settings: the LM22 signature matrix was used, consisting of 547 genes to distinguish 22 mature immune cell populations; B-mode batch correction was used; quantile normalization was disabled; 1000 permutations were performed for significance analysis. Only CIBERSORTx results with a p-value < 0.05 were included in subsequent analyses. To increase abundance of more comprehensive immune cell phenotypes, immune cell fractions obtained through LM22 were aggregated as outlined in “Aggregate 2” of the **Supplementary Materials** in Thorsson et al. (75) to obtain 9 immune cell aggregate phenotypes. Differences in immune cell infiltration proportions were analyzed using Mann-Whitney tests.

### 2.4 Dirichlet regression and statistical analysis

As the output from CIBERSORTx is considered compositional data that carries relative information as proportions of the total amount of immune cell infiltration, summing to 1 for each sample, traditional data analysis may violate modeling assumptions, such as homoscedasticity (76, 77). Therefore, Dirichlet regression was also performed comparing immune cell fractions between groups of interest using DirichletReg v0.7-1 (76), R version 4.3.0 (78) using the common parametrization model. The regression estimate coefficients obtained using this method can be interpreted similarly to odds ratios if taken as exponentiated coefficients (76). Differences in clinical characteristics of patients were analyzed using Chi-square test or ANOVA, where appropriate. Survival probabilities for each RECIST group were estimated using the Kaplan-Meier method and the log-rank test. Univariate cox proportional hazards for immunomodulators was performed in R using the survival package v3.5-5 (79). Visualization was performed using GraphPad PRISM v9.5.0 and R 4.3.0.

## 3 Results

### 3.1 Clinical parameters of included PDAC patients from the COMPASS trial

In total, 145 of the 195 patients from the COMPASS trial dataset met inclusion criteria for our secondary analysis (**Table 1**). Compared with patients who received Gem-based therapy, mFFX treated patients were significantly younger, predominantly male, and were more likely to have locally advanced disease rather than metastatic disease. However, there was no statistically significant difference in terms of site of biopsy or chemotherapy response between patients receiving mFFX and Gem-based therapy. Similar to previous studies in the efficacy standard chemotherapy regimens in PDAC (3, 64), we observed an improvement in median overall survival (OS) in patients who received FOLFIRINOX compared to Gem-based therapy, although this did not reach significance (median OS 307 vs 254 days, p-value = 0.12, **Supplemental Figure 1**). For the entire cohort, chemotherapy response correlated with overall survival (**Figure 1B**, p-value < 0.0001), similar to previous studies utilizing RECIST in a metastatic PDAC setting (80, 81). As expected, patients with tumors that progressed or demonstrated no change with treatment (nonresponder) had significantly shorter median survival compared to the patients with tumors that decreased in size following treatment (responder) (199 vs 359 days).

**Table 1:** Clinical characteristics of patients from the COMPASS trial included in analysis.

CIBERSORT Cohort	All	mFFX	Gem-based	P-value
**# Included**	145	83	62	
Age				
Mean (SD)	62.7 (9.3)	60.0 (8.6)	66.0 (9.2)	**<0.0001**
Median [Min, Max]	64 [29, 84]	61 [35, 77]	67 [29, 84]	
Gender (%)				
M	81 (55.8)	54 (66.7)	27 (33.3)	**0.0099**
F	64 (44.2)	29 (45.3)	35 (54.7)	
Disease Status (%)				
Locally Advanced	20 (13.8)	16 (80.0)	4 (20.0)	**0.0267**
Metastatic	125 (86.2)	67 (53.6)	58 (46.4)	
Biopsy Site (%)				
Pancreas	46 (31.7)	25 (54.3)	21 (45.7)	0.6312
Liver	99 (68.3)	58 (58.6)	41 (41.4)	
Therapy Response (%)				
Responders	94	52	42	0.4820
Nonresponders	50	31	19	

Bolded values are those with p-values below a cutoff of 0.05, suggesting significance.

### 3.2 Tumor site-specific variations in the PDAC tumor immune microenvironment

**Figure 2:**
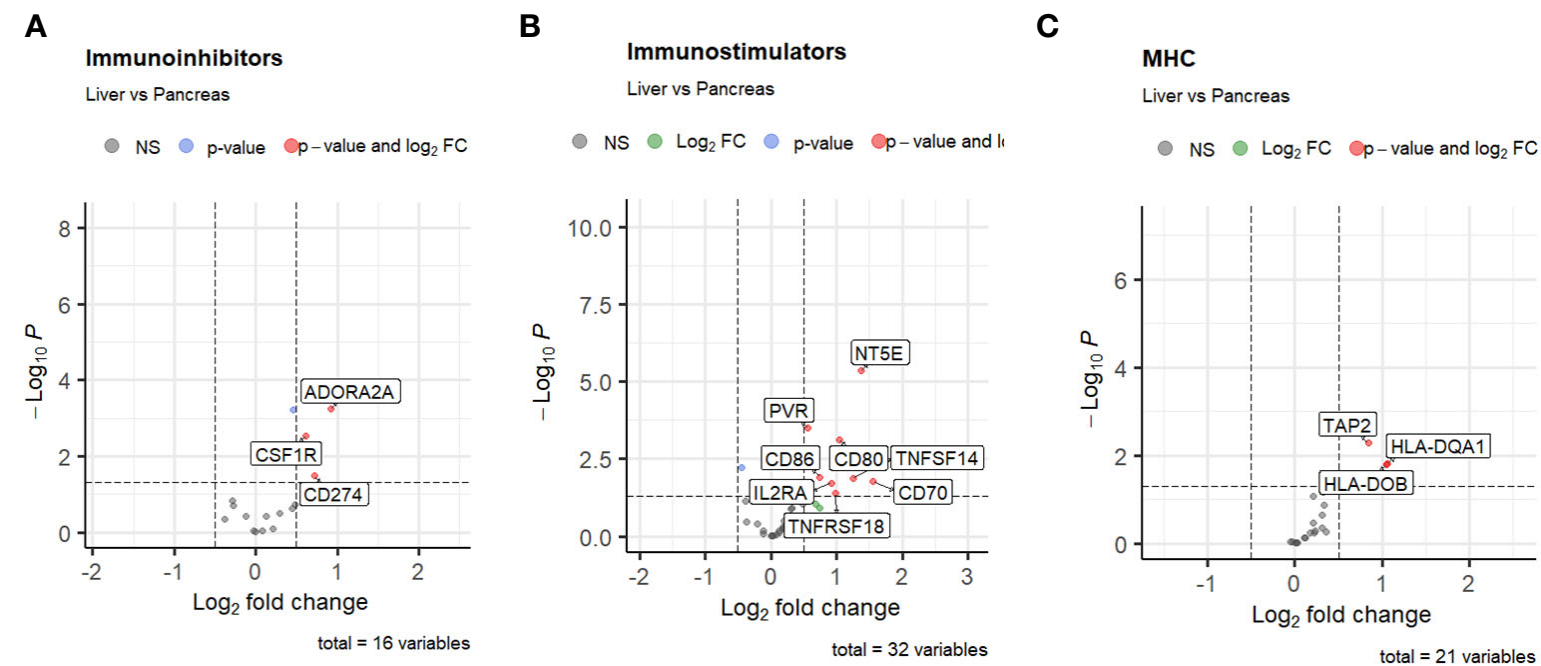
Volcano plots of differential expression of **(A)** immunoinhibitors, **(B)** immunostimulators, and **(C)** MHC genes from TISIDB based on site of biopsy. The threshold for log_2_ fold change is set at 0.5, and the threshold for false discovery rate is set at 0.05.

Considering the unique pre-treatment patient samples within our cohort, we initially sought to determine whether site-specific differences existed in the cellular components of the PDAC TiME (68) between primary pancreatic tumor biopsies and metastatic liver samples. Compared with pancreatic tumor samples, metastatic liver biopsies had significantly higher expression of multiple immunomodulatory genes. Immunoinhibitory genes such as ADORA2A (log fold-change 0.92, p-value < 0.0001) (82, 83), CSF1R (log fold-change 0.62, p-value = 0.003) (84, 85), and CD274/PD-L1 (log fold-change 0.72, p-value 0.03) had significantly higher expression in the liver biopsy samples compared with pancreatic samples (**Figure 2**). We also noted differences in immunostimulatory and MHC genes based on site of biopsy. A total of 12 immunostimulatory genes within the annotation (CD70, CD80, CD86, CD276, IL2RA, MICB, NT5E, PVR, TNFSF14, TNFRSF14, TNFRSF18, ULBP1) were significantly differentially expressed between liver and pancreatic biopsy samples. Of these, only TNFRSF14, a membrane-bound receptor (86) with both pro-inflammatory and anti-inflammatory immune signaling pathways (87), was downregulated in the liver biopsy samples compared to the pancreatic biopsy samples (log fold-change -0.43, p-value = 0.003), while the other immunostimulatory genes were upregulated. Similarly, of the MHC genes that were significantly differentially expressed (TAP2, HLA-DOB, TAP1, HLA-DQA1), all were upregulated in the liver biopsy samples compared to the pancreatic biopsy samples. Taken together, this data suggests that the TiME of liver metastases in PDAC undergoes more dynamic regulation compared to that of primary pancreatic lesions.

### 3.3 Pre-treatment PDAC TiME differences associated with chemotherapy response

**Figure 3:**
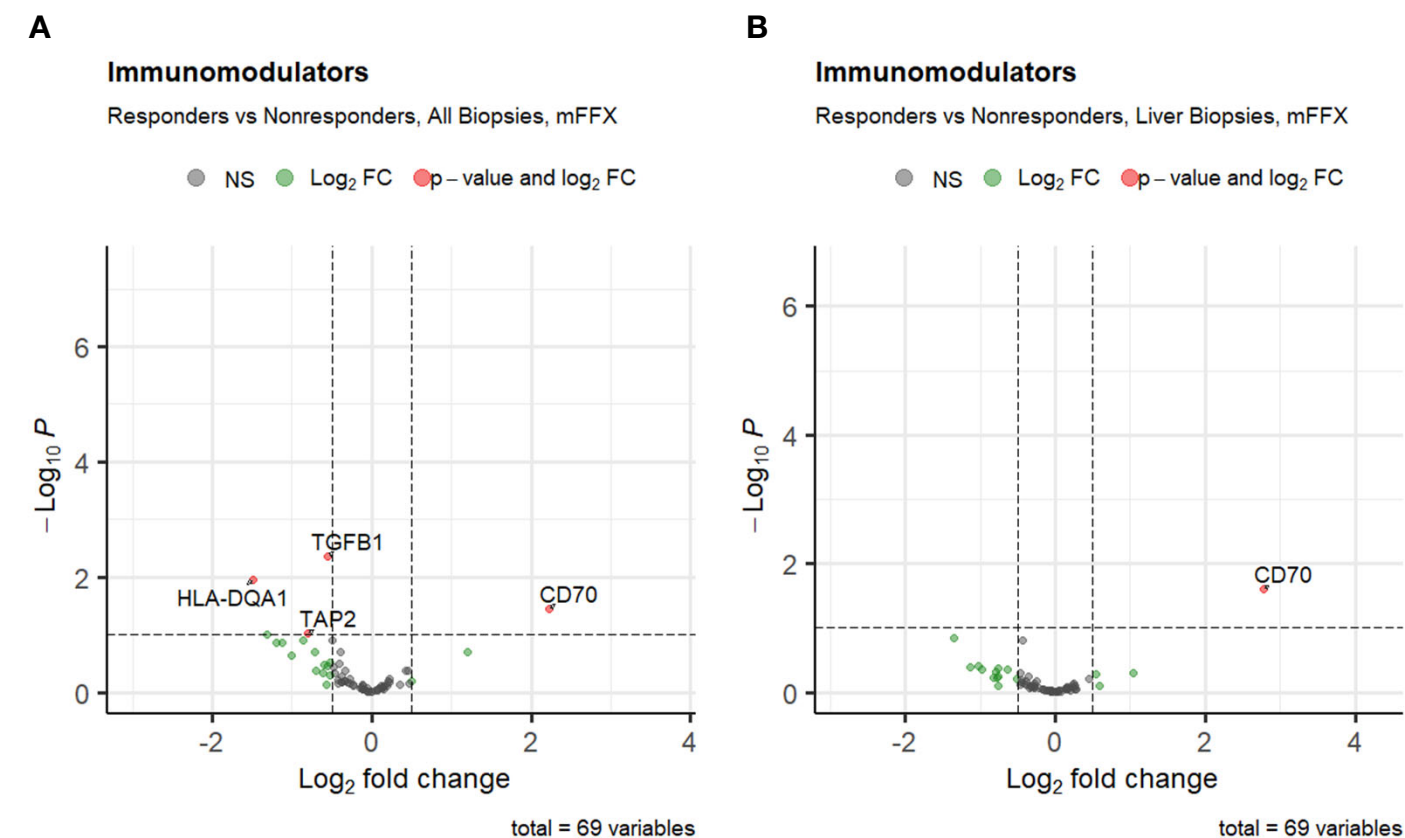
Volcano plots of differential expression of immunomodulatory genes from TISIDB for **(A)** patients who received mFFX, and **(B)** patients who received mFFX and had metastatic liver biopsies.

Next, we investigated the association of pre-treatment PDAC tumor immune cell infiltration with response to either mFFX or Gem-based therapy. We first compared the initial immune cell infiltration of each treatment group to determine if there were upfront differences in immune populations that may bias downstream analyses. While patients who received mFFX had decreased infiltration by the CD4 T cell aggregate compared to the Gem-based group, there were no statistical differences in immune infiltration by the individual CD4 T cell phenotypes included in the aggregate (**Supplementary Table 1**), suggesting a similar initial immune cell phenotypic infiltration between treatment groups.Chemotherapy response was associated with variations in expression of tumor pre-treatment immunomodulating genes (**Figures 3A, B**). The immunoinhibitory gene TGFB1 was downregulated in responders to mFFX compared to nonresponders (log fold-change -0.55, p-value < 0.005), while the immunostimulator CD70 (88) was significantly upregulated (log fold-change 2.2, p-value < 0.05). In the metastatic liver-cohort, only the upregulation of CD70 remained significantly increased among responders (log fold-change 2.8, p-value < 0.05). There were no significantly upregulated or downregulated genes associated with Gem-based therapy response (**Supplementary Figures 2A, B**).

### 3.4 Clinical impact of immunomodulatory genes between biopsy sites

**Figure 4:**
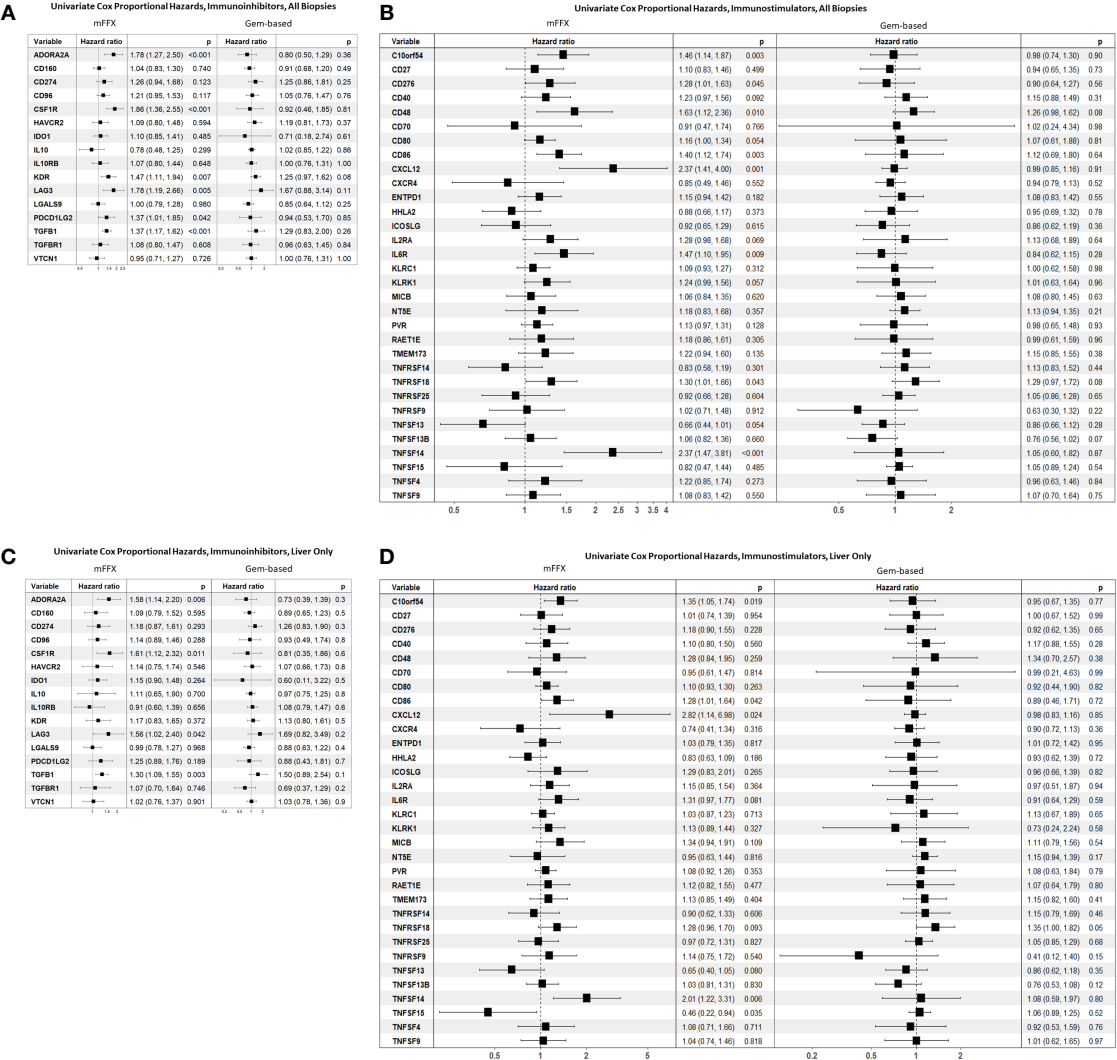
Forest plot of univariate Cox proportional hazard analyses based on chemotherapy received. **(A)** Reports hazard ratios for immunoinhibitors in all biopsies, **(B)** reports hazard ratios for immunostimulators in all biopsies. **(C)** Reports hazard ratios for immunoinhibitors in metastatic liver biopsies, and **(D)** reports hazard ratios for immunostimulators in metastatic liver biopsies.

We wanted to evaluate the impact of immunomodulator gene expression on clinical outcomes within our patient cohort. As therapy response strongly correlated with overall survival in this patient cohort from the COMPASS trial, we investigated the association between immunomodulatory gene expression and survival in the different therapy cohorts based on treatment response (**Figures 4A-D**). Across all biopsies, the immunoinhibitors ADORA2A, CSF1R, KDR/VEGFR2, LAG3, PDCD1LG2, and TGFB1 were significantly associated with worse survival in patients who received mFFX (**Figures 4A, C**). A subset of these immunoinhibitors including ADORA2A, CSF1R, LAG3, and TGFB1 were also significantly associated with worse survival in the subset of liver biopsies from patients who received mFFX. There were no immunoinhibitors associated with either improved or worsened survival in the Gem-based cohort for all samples and the liver subset.

Multiple immunostimulatory genes were also associated with worse survival in the mFFX cohort across all samples (**Figure 4B**), including C10orf54, CXCL12, and TNFSF14/LIGHT. While there was an overlap in significant immunostimulators between all samples and the liver sample subset (**Figure 4D**), TNFSF15/TL1A was only significantly associated with improved survival in liver samples (HR 0.46, p-value = 0.03). Again, we found no immunostimulatory genes that were significantly associated with response to Gem-based therapy in either all samples or the liver subset.

### 3.5 Quantification of immune cell infiltration using CIBERSORTx

Similar to our immunomodulatory analysis, we observed significant differences using CIBERSORTx in infiltrating immune cell proportions on comparison of the primary lesion pancreatic samples compared with metastatic liver biopsies (**Table 2**). Metastatic liver samples had both increased macrophage (1.2 times more, p-value < 0.05) and increased eosinophil infiltration (1.4 times more, p-value < 0.05) compared to pancreatic biopsies from the primary lesion. Further analysis of the specific macrophage phenotypes revealed an increased M2 macrophage fraction in the liver samples, indicating a more immunosuppressed metastatic TiME compared to the primary lesion.

**Table 2:** Comparison of immune infiltration of liver vs pancreas biopsies.

Liver vs Pancreas			
Immune Cell Phenotype	Odds Ratio	CI	P-value
B cells	1.06	0.84 - 1.28	0.613
T cell CD8	1.03	0.79 - 1.26	0.816
T cell CD4	1.01	0.75 - 1.27	0.955
NK cells	1.09	0.85 - 1.33	0.472
Macrophages	1.23	1.03 - 1.43	**0.043**
Dendritic cells	1.15	0.92 - 1.39	0.239
Mast cells	1.11	0.91 - 1.31	0.313
Eosinophils	1.48	1.17 - 1.79	**0.013**
Neutrophils	1.24	0.94 - 1.55	0.161
Macrophages M0	1.54	1.17 - 1.90	**0.022**
Macrophages M1	1.14	0.78 - 1.49	0.487
Macrophages M2	1.69	1.32 - 2.06	**0.005**

Bolded values are those with p-values below a cutoff of 0.05, suggesting significance.

For the entire cohort of treated patients, increased infiltration by 8 out of the 9 immune cell aggregate phenotypes except NK cells were significantly associated with response to mFFX treatment. Pre-treatment tumor immune cell populations were not associated with response to Gem-based treatment (**Table 3A**). On subset analysis of PDAC patients with liver metastasis, only increased pre-treatment CD8 T-cell, dendritic cell, and neutrophil infiltration were only significantly associated with therapy response to mFFX (**Table 3B**). Conversely, increased total mast cell infiltration was only associated with response to Gem-based therapy in the liver TiME.

**Table 3A:** Comparison of immune infiltration in responders versus nonresponders for patients treated with mFFX and Gem-based therapy.

Responders vs Nonresponders	mFFX (n = 83)			Gem-based (n = 62)		
Immune Cell Phenotype	Odds Ratio	CI	P-value	Odds Ratio	CI	P-value
B cells	1.40	1.12- 1.68	**0.018**	1.10	0.76 - 1.43	0.587
T cell CD8	1.56	1.25 - 1.86	**0.004**	1.30	0.94 - 1.66	0.152
T cell CD4	1.46	1.12 - 1.80	**0.030**	1.12	0.73 - 1.51	0.560
NK cells	1.26	0.96 - 1.57	0.135	0.97	0.61 - 1.33	0.867
Macrophages	1.41	1.16 - 1.67	**0.008**	1.14	0.84 - 1.45	0.387
Dendritic cells	1.48	1.18 - 1.79	**0.011**	1.13	0.77 - 1.50	0.495
Mast cells	1.40	1.15 - 1.66	**0.009**	1.34	1.03 - 1.65	0.063
Eosinophils	1.57	1.18 - 1.96	**0.023**	0.99	0.54 - 1.45	0.974
Neutrophils	1.56	1.18 - 1.94	**0.023**	1.23	0.76 - 1.70	0.383

Bolded values are those with p-values below a cutoff of 0.05, suggesting significance.

**Table 3B:** Comparison of immune infiltration in responders versus nonresponders for patients treated with mFFX and Gem-based therapy, metastatic liver biopsies only.

Responders vs Nonresponders	Liver_mFFX (n = 58)			Liver_Gem-based (n = 41)		
Immune Cell Phenotype	Odds Ratio	CI	P-value	Odds Ratio	CI	P-value
B cells	1.31	0.98 - 1.64	0.104	1.24	0.86 - 1.63	0.267
T cell CD8	1.57	1.21 - 1.92	**0.013**	1.33	0.91 - 1.75	0.181
T cell CD4	1.36	0.95 - 1.76	0.142	1.28	0.84 - 1.72	0.272
NK cells	1.25	0.89 - 1.60	0.228	1.11	0.69 - 1.54	0.616
Macrophages	1.24	0.94 - 1.54	0.156	1.32	0.97 - 1.68	0.123
Dendritic cells	1.48	1.12 - 1.83	**0.032**	1.33	0.91 - 1.74	0.182
Mast cells	1.33	1.03 - 1.63	0.061	1.61	1.25 - 1.97	**0.010**
Eosinophils	1.55	1.10 - 2.00	0.057	1.33	0.81 - 1.86	0.279
Neutrophils	1.69	1.25 - 2.14	**0.020**	1.49	0.94 - 2.03	0.151

Bolded values are those with p-values below a cutoff of 0.05, suggesting significance.

Taken together, these data suggest that pre-treatment infiltration by different immune cell phenotypes differs based on site and are associated with therapy response in both a site-specific and therapy-specific fashion. Response to mFFX was more associated with increased infiltration by CD8 T-cells compared to Gem-based regimens, indicating that the pre-treatment TiME may be more impactful for patients receiving mFFX.

## 4 Discussion

Across cancers, the TiME is a well described mediator of patient survival and can influence treatment response (89). A growing body of evidence supports the ability of FOLFIRINOX to augment tumoral immunity. However, the influence of pre-treatment TiME on FOLFIRINOX response is not well described. Insight into tumor microenvironment features associated with chemotherapy response may help to identify key mediators of efficacy and point to future opportunities in designing next generation chemo-ICI strategies. Utilizing access to a unique dataset of treatment-naïve biopsy samples of advanced or metastatic PDAC patients from the COMPASS trial, we hypothesized that the components of the pre-treatment TiME would differ between metastatic and primary lesions and would be associated with therapy response and survival in a cohort of advanced PDAC patients.

In the present study, we identified key site-specific differences in the cellular and genomic components of the PDAC TiME. Our analysis identified that PDAC metastatic liver biopsies had more variable expression of immunomodulatory genes compared to pancreas biopsies. Multiple immunoinhibitory genes such as CSF1R (84, 85) and components of immune checkpoint signaling pathways such as CD86-CTLA4 (90), PD-1/PD-L1 (91), and PVR-TIGIT (92) were upregulated in liver metastatic samples. Notably, no immunomodulatory genes were identified that were significantly upregulated in the primary lesion TiME relative to the metastatic tumor samples. Compared to the TiME of primary lesion pancreatic biopsies, the metastatic liver biopsies also demonstrated increased infiltration by M0 and M2 macrophages. M0 macrophages are considered undifferentiated macrophages that can be polarized into different functional phenotypes such as M1 and M2 (93). However, a growing body of evidence suggests that M0 macrophages are not a benign member of the TiME. They are associated with worse outcomes in multiple cancers such as breast (94), prostate (95), and lung cancer (96), and possess a transcriptional profile similar to that of M2 macrophages (97, 98). This, combined with an increased infiltration of the immunoinhibitory M2 macrophage (46–52) and the immunomodulatory findings stated above, suggests that the metastatic liver TiME is more dynamically regulated and overall immunosuppressed compared to the TiME of the primary lesion.

An inflamed pre-treatment TiME has been recognized as a predictor of response to neoadjuvant chemotherapy in various cancers (99, 100). One of the earlier markers used to predict response was the systemic immune-inflammatory index (SII) (101), based on peripheral neutrophils, platelets, and lymphocytes that portended survival in NSCLC (102), gastric (103, 104), and colorectal (105) cancer. Similarly, the Immunoscore, which was initially developed in colorectal cancer (106) and is based on CD3+/CD8+ lymphocyte quantification in tumors, predicts disease-free survival and overall survival in colorectal cancer (54, 55) and other cancers (56, 57). Notably, standard chemotherapy regimens for colorectal cancer have significant overlapping antineoplastic agents with FOLFIRINOX, including FOLFOX, XELOX, FOLFIRI, FOLFOXIRI, or CAPIRI (107), implying that a pre-treatment immune contexture can impact therapy response in PDAC as well. This is supported by retrospective studies in PDAC, which have demonstrated associations between survival and various pre-treatment TIL populations. For example, increased CD8 TIL presence correlated with improved survival (108, 109), while M2 macrophage infiltration correlated with worsened survival (110–112). However, limited data exist in PDAC directly addressing the effect of the pre-treatment immune contexture on chemotherapy. In this study, we show that key members of the pre-treatment TiME are also significantly associated with treatment response. Notably, increased infiltration by mutually exclusive immune cell phenotypes were associated with response to different chemotherapeutic regimens. Increased CD8 T-cell infiltration was significantly associated with tumor response to mFFX in the entire cohort and on subset analysis of patients with liver metastasis. Previous reports have demonstrated that mFFX treatment is associated with an increased infiltration of CD8+ T cells and reduced Tregs (41–43, 113) suggesting that mFFX can augment the PDAC TiME. However, our analysis demonstrates that the pre-treatment CD8 T-cell infiltration status of the PDAC TiME may also impact response to FOLFIRINOX. This opens the question of whether the observed increase in CD8+ T cells post-mFFX and the associated favorable response were due to the presence of a high CD8+ T cell population pre-treatment. Our findings implicate the tumor immune status and favorable biology of the treatment-naive tumor may influence response. It is highly likely that both the pre-treatment TiME and chemotherapy-induced CD8+ T cells contribute to a favorable response with mFFX. In contrast, Gem-based regimens were not associated with the presence of any immune cell population across all samples, and only mast cell aggregates in the liver subset. While mast cell infiltration is typically associated with tumor growth (114, 115), higher mast cell infiltration was significantly correlated with overall survival and response to gemcitabine in a cohort of biliary tract cancer patients (116).

These treatment-specific patterns in the TiME were also seen when analyzing immunomodulators associated with therapy response and survival. For example, the immunoinhibitory and pro-tumorigenic (117) cytokine TGFB1 was significantly downregulated in responders and associated with worse survival in the mFFX treatment cohort. Other genes such as ADORA2A, CSF1R, and LAG3 were significantly associated with worse survival in patients in the mFFX cohort across all samples and liver-only samples. No immunomodulatory genes were associated with therapy response in the Gem-based therapy cohort when comparing either all biopsies or focusing solely on liver biopsy samples (**Supplementary Figures S2A, B**). Similarly, no immunomodulatory genes were associated with survival within the Gem-based cohort (**Figures 4A–D**). In the context of pre-clinical studies which demonstrate that FOLFIRINOX (18–20) and gemcitabine (118, 119) impact tumor immunity, our data suggest that the interactions between the cellular and genomic components of the pre-treatment PDAC TiME with FOLFIRINOX and gemcitabine may be mechanistically different.

As reflected within this patient cohort, overall survival for patients with advanced stage PDAC remains abysmal, with 5-year survival of less than 10% (2) and highlights the need for strategies to improve outcomes for this large subset of PDAC patients. Currently, aside from the patient’s performance status there are no indications for administering one regimen over the other (41, 42). Our data suggest that FOLFIRINOX-based chemotherapy approach may be advantageous in select patients with a favorable pre-existing TiME. In such patients, and in patients of borderline performance status that may sway a clinician against the use of FOLFIRINOX (41, 42), an immune-based indication for chemotherapy may provide a more nuanced approach to cancer therapy and improve patient outcomes. Furthermore, the implication of a dynamically regulated, immunosuppressed metastatic TiME in PDAC suggests potential avenues for targeted therapies and implies the need to incorporate stage of disease into future design of immune-targeted PDAC therapeutic strategies. For example, the presence of multiple upregulated immune checkpoint pathways in the metastatic PDAC TiME may be targeted via existing checkpoint blockade therapies (84, 120–122) and may represent a potential therapy target to improve outcomes for patients with metastatic PDAC. Another consideration would be to capitalize on the impact of a pre-treatment TiME on chemotherapy through immunological priming. For example, preclinical studies show administration of immunomodulatory cytokines such as interferon sensitize PDAC cell lines to gemcitabine (123, 124). Another strategy could combine chemotherapy with the prior use of mRNA vaccines to expand tumor-specific T-cells (45). As multiple immunomodulatory genes were only associated with survival in the mFFX cohort, adjunctive immunotherapeutic strategies could improve mFFX response in either a chemotherapy-only or chemo-ICI regimen. Potential candidates include the use of antibodies or bispecific molecules to target TGFB, which are in clinical testing (125). Agents to block CSF1R, such as surufatinib, are also in testing, and have demonstrated anti-tumoral efficacy in phase III trials (126).

Limitations exist within this study. The COMPASS trial included only patients with advanced or metastatic PDAC, and the results may not be valid in a stage I/II, resectable cohort. CIBERSORTx only provides relative proportions of immune cell infiltration and may not be reflective of absolute infiltration especially for inter-sample comparison (127); as such, we are unable to quantify how potential differences in the absolute immune infiltration may impact chemotherapy response, and will require further study. Additionally, the LM22 signature matrix used in deconvolutional analysis was derived from microarray-derived data from PBMCs (128) and may not be reflective of the totality of immune cell phenotypes within the PDAC TiME. However, this study utilized a large, unique dataset of treatment-naïve PDAC samples to analyze the TiME; future efforts would investigate the *in silico* analysis in both *in vitro* and *in vivo* PDAC models to validate the results and advance our understanding of the PDAC tumor biology.

## Data availability statement

Publicly available datasets were analyzed in this study. This data can be found here: https://ega-archive.org/datasets/EGAD00001004548.

## Ethics statement

The studies involving humans were approved by Institutional Review Board approval and written consent for the COMPASS trial was obtained from participating institutions (University Health Network, Toronto, Ontario, Canada; MUHC Centre for Applied Ethics, Montreal, Quebec, Canada; and Queen’s University Health Sciences and Affiliated Teaching Hospitals Research Ethics Board, Kingston, Ontario, Canada) and a data use agreement was completed by Baylor College of Medicine with the Ontario Institute for Cancer Research for use of the data within this study. The studies were conducted in accordance with the local legislation and institutional requirements. The participants provided their written informed consent to participate in this study.

## Author contributions

ZG: Conceptualization, Data curation, Formal Analysis, Investigation, Methodology, Software, Visualization, Writing – original draft, Writing – review & editing. SK: Data curation, Investigation, Software, Writing – original draft, Writing – review & editing. DE: Supervision, Writing – review & editing. JA: Writing – review & editing. GV: Writing – review & editing. WF: Writing – review & editing. ZS: Methodology, Supervision, Writing – review & editing. MR: Writing – review & editing. HL: Data curation, Software, Supervision, Writing – review & editing. EC: Conceptualization, Data curation, Funding acquisition, Project administration, Resources, Supervision, Writing – review & editing.

## Supplementary material

Supplementary Figure 1 Kaplan-Meier estimate of overall survival of patients who received mFFX versus Gem-based therapy.

Supplementary Figure 2 Volcano plots of differential expression of immunomodulatory genes from TISIDB for **(A)** patients who received Gem-based therapy, and **(B)** patients who received Gem-based therapy and had metastatic liver biopsies.

Supplementary Table 1 Comparison of immune infiltration of mFFX versus Gem-based therapy.
