# Supplementary figures and images for: Pre-treatment inflamed tumor immune microenvironment is associated with FOLFIRINOX response in pancreatic cancer
Source: Front Oncol. 2023 Nov 23;13:1274783. doi: 10.3389/fonc.2023.1274783 (PMC10701674; doi:10.3389/fonc.2023.1274783)

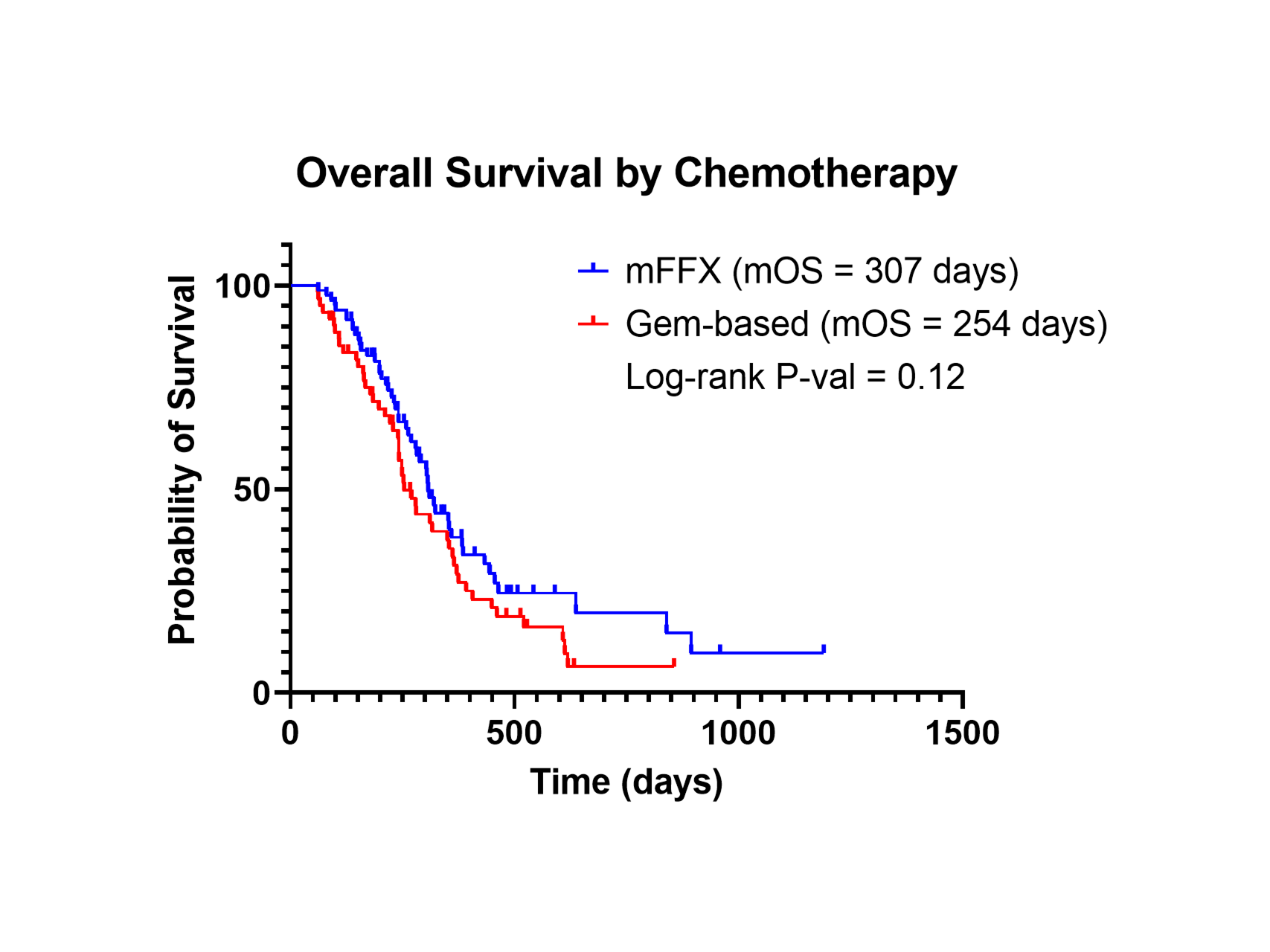

Supplement: Supplementary Figure 1 — Kaplan-Meier estimate of overall survival of patients who received mFFX versus Gem-based therapy. [file Image_1.tif]

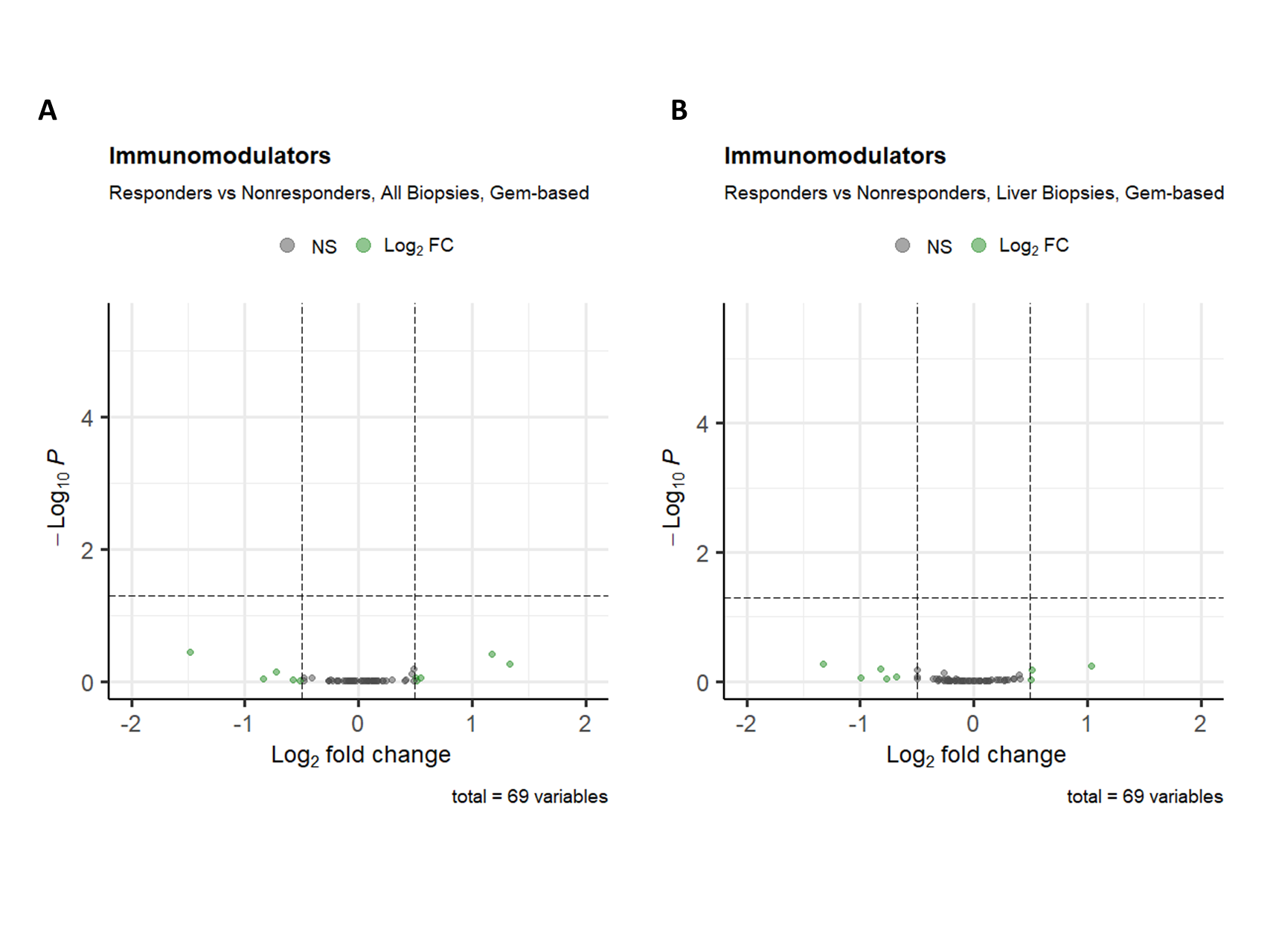

Supplement: Supplementary Figure 2 — Volcano plots of differential expression of immunomodulatory genes from TISIDB for (A) patients who received Gem-based therapy, and (B) patients who received Gem-based therapy and had metastatic liver biopsies. [file Image_2.tif]
