# Supplementary material for: Pre-treatment inflamed tumor immune microenvironment is associated with FOLFIRINOX response in pancreatic cancer
Source: Front Oncol. 2023 Nov 23;13:1274783. doi: 10.3389/fonc.2023.1274783 (PMC10701674; doi:10.3389/fonc.2023.1274783)
Supplement: Supplementary Table 1 — Comparison of immune infiltration of mFFX versus Gem-based therapy. [file Table_1.docx]

**Supplemental Table 1**: Comparison of immune infiltration of mFFX versus Gem-based therapy.

| **mFFX vs Gem-based** |  |  |  |
| --- | --- | --- | --- |
| **Immune Cell Phenotype** | **Odds Ratio** | **CI** | **P-value** |
| B cells | 0.90 | 0.69 - 1.10 | 0.295 |
| T cell CD8 | 0.85 | 0.63 - 1.07 | 0.136 |
| T cell CD4 | 0.72 | 0.47 - 0.96 | **0.007** |
| NK cells | 0.95 | 0.73 - 1.17 | 0.660 |
| Macrophages | 0.86 | 0.67 - 1.04 | 0.104 |
| Dendritic cells | 0.96 | 0.74 - 1.18 | 0.725 |
| Mast cells | 0.96 | 0.77 - 1.15 | 0.667 |
| Eosinophils | 0.84 | 0.56 - 1.12 | 0.229 |
| Neutrophils | 1.03 | 0.75 - 1.32 | 0.813 |
| T cell CD4 Naïve | 1.03 | 0.68 - 1.38 | 0.879 |
| T cell CD4 Mem Resting | 0.99 | 0.56 - 1.42 | 0.981 |
| T cell CD4 Mem Activated | 0.96 | 0.60 - 1.32 | 0.815 |
